# Characterization of nano-structural and nano-mechanical properties of osteoarthritic subchondral bone

**DOI:** 10.1186/s12891-016-1226-1

**Published:** 2016-08-24

**Authors:** Qiliang Zuo, Shifeier Lu, Zhibin Du, Thor Friis, Jiangwu Yao, Ross Crawford, Indira Prasadam, Yin Xiao

**Affiliations:** 1 Ministry Education Key Laboratory for Oral Biomedical Engineering, School of Stomatology, Wuhan University, Wuhan, 430079 People’s Republic of China; 2 Xiamen Dental Hospital, Xiamen, Fujian Province China; 3 Institute of Health and Biomedical Innovation, School of Chemistry, Physics, Mechanical Engineering, Queensland University of Technology, Brisbane, Australia; 4 Orthopedic Department, Prince Charles Hospital, Brisbane, Australia; 5 Institute of Health and Biomedical Innovation, Queensland University of Technology, Kelvin Grove Campus, Brisbane, Qld 4059 Australia

**Keywords:** Osteoarthritis, Subchondral bone, Nano-structure, Crystallinity, Ca/P, Bone hierarchical structure

## Abstract

**Background:**

Although articular cartilage is the primary tissues affected by osteoarthritis (OA), the underlying subchondral bone also undergoes noticeable changes. Despite the growing body of research into the biophysical and mechanical properties of OA bone there are few studies that have analysed the structure of the subchondral sclerosis at the nanoscale. In this study, the composition and nano-structural changes of human osteoarthritis (OA) subchondral bone were investigated to better understand the site-specific changes.

**Methods:**

OA bone samples were collected from patients undergoing total knee replacement surgery and graded according to disease severity (grade I: mild OA; grade IV: severe OA). Transmission electron microscopy (TEM), Electron Diffraction, and Elemental Analysis techniques were used to explore the cross-banding pattern, nature of mineral phase and orientation of the crystal lattice. Subchondral bone nano-hydroxyapatite powders were prepared and characterised using high resolution transmission electron microscopy (HR-TEM) and fourier transform infrared spectroscopy (FTIR). Subchondal bone mechanical properties were investigated using a nano-indentation method.

**Results:**

In grade I subchondral bone samples, a regular periodic fibril banding pattern was observed and the *c*-axis orientation of the apatite crystals was parallel to the long axis of the fibrils. By contrast, in grade IV OA bone samples, the bulk of fibrils formed a random and undulated arrangement accompanied by a circular oriented pattern of apatite crystals. Fibrils in grade IV bone showed non-hierarchical intra-fibrillar mineralization and higher calcium (Ca) to phosphorous (P) (Ca/P) ratios. Grade IV OA bone showed higher crystallinity of the mineral content, increased modulus and hardness compared with grade I OA bone.

**Conclusions:**

The findings from this study suggest that OA subchondral sclerotic bone has an altered mineralization process which results in nano-structural changes of apatite crystals that is likely to account for the compromised mechanical properties of OA subchondral bones.

**Electronic supplementary material:**

The online version of this article (doi:10.1186/s12891-016-1226-1) contains supplementary material, which is available to authorized users.

## Background

Osteoarthritis (OA) is a leading cause of disability and joint dysfunction in adults. Since the predominant feature of OA is degeneration of articular cartilage, most studies into the pathogenesis of OA have tended to focus on the mechanisms involved in the destruction of the articular cartilage. However, subchondral bone sclerosis is also a well-characterized manifestation in OA and many studies have emphasized the importance of subchondral bone changes, such as composition, architecture, quality, and regulation as important distinguishing features of OA [1–3]. Studies have demonstrated abnormal biochemistry in subchondral bone in OA compared to normal controls, with increased bone formation and relatively high bone mineral density (BMD) [3, 4]. It is well known that changes to the composition of the subchondral bone matrix in OA are associated with alterations in bone microarchitecture. During the end stage of OA, microarchitectural characteristics of the subchondral bone are (i) thickening of subchondral bone plate and trabecular bone, (ii) increased bone volume fraction, (iii) decrease of trabecular separation and bone marrow spacing, (iv) and transformation of the trabeculae from a rod-like to a plate-like configuration [5]. Disordered microarchitecture within the subchondral bone causes it to become relatively stiffer and denser in OA affected bone and leads to a disruption of the equilibrium of the mechanical loading between cartilage and subchondral bone. Although sclerotic bone is less well mineralized, it suffers greater absorption of local stresses, reducing load transmission to the deeper subarticular region and resulting in OA progression [6].

Despite the growing body of research into the biophysical and mechanical properties of OA bone [7, 8] there are few studies that have analysed the structure of the subchondral sclerosis at the nanoscale. It is therefore not well understood how the hypomineralized subchondral sclerosis region responds to the increased mechanical strains. The hierarchical structure of bone, from nano scale to the organ level, ultimately determines its mechanical strength and properties. At the nano-scale, bone is a composite with a quasiperiodic structure, consisting of carbonated hydroxyapatite (HA) crystals, which are embedded into collagen fibrils. An exact match of collagen fibrils and mineral crystal organization provides bone with its capacity to withstand mechanical loads. Until now, the evaluation of OA includes an assessment of a patient’s bone mineral density (BMD), using techniques such as computer tomography (CT, or micro-CT) and magnetic resonance imaging (MRI). Changes seen using micro-CT (μCT) are morphometric parameters, such as bone volume fraction and trabecular number, thickness, and separation. However, these techniques do not provide an understanding of mechanical properties such as the hardness, modulus, and toughness of the tissue and the quality of mineral and fibres at nano-scale, all of which are independent of bone mass or micro-architecture.

In this study we evaluated the subchondral bone structure at various length-scales in two representative Polar Regions, with and without sclerosis (grade IV OA and grade I OA), in patient matched samples with an aim of providing a better understanding of the structural and compositional determinants of bone strength. For this purpose we have used advanced imaging techniques to characterize the material quality of the OA bone and its mechanical strength at the nano-scale level. Nanoindentation was used to determine hardness and elastic modulus at defined local positions of sub-micrometer sizes in various subchondral bone and trabecular areas. Transmission electron microscopy (TEM) imaging, Electron Diffraction, and Elemental Analysis techniques were used to explore bone fibrils banding patterns, the nature of the mineral phase and the orientation of crystal lattices. Furthermore, subchondral bone nano-hydroxyapatite powders were prepared and characterised using high-resolution transmission electron microscopy (HR-TEM) and Fourier transform infrared spectroscopy (FTIR). Applying these techniques, we found that severe OA-affected bone had altered nano-structural and mechanical properties.

## Methods

### Study subjects

Ten grade I and ten grade IV OA samples were collected from age-matched and sample-matched OA patients (grade IV OA in medial compartment and grade I OA in distal compartment of the same patient’s OA knee as outlined below) undergoing total knee replacement surgery. The patients were recruited for this study (mean age 57.1 ± 6.3 years) after the obtaining of informed consent from each participant. All OA patients had radiographic evidence of grade IV OA, according to the Kellgren and Lawrence criteria [9, 10]. Tibial plateaus were marked as medial and lateral compartments and were labelled with surgical marker at the anterior end of the tibia plateau, and inferior end for future orientation references. Patients with any bone disorders other than OA, or reported conditions that affect bone metabolism, or receiving treatment that affects bone metabolism such as anti-resorptive drugs, or hormonal replacement therapy, were excluded from the study. The study protocol was approved by the Human Research Ethics committees of the Queensland University of Technology and Prince Charles Hospital.

### Subchondral bone specimen preparation

Each tibial plateau was visually sectioned into two categories taking into account the sclerosis of the trabecular bone and degeneration of the articular cartilage: (1) non-sclerotic tissue with intact cartilage, (2) severely sclerotic tissue and moderate cartilage degeneration with partial exposure of subchondral bone. Then the grade I OA group and grade IV OA group were further selected according to previous studies [9, 10] and the sample classification was aided by histopathology grading system as a Mankin score. All the samples were divided into grade I OA group (relative normal bone with a Mankin score less than 3) and grade IV OA group (severe damaged and sclerotic bone with a Mankin score greater than 12) [11–13]. A total of 10 cylindrical bone blocks with a diameter of 5 mm, including osteochondral and adjacent subchondral bone, were prepared from all the visual grades. All specimens were fixed in 4 % paraformaldehyde (PFA) and scanned by micro CT (Scanco 40, Switzerland) with isotropic voxel size of 18 μm, using 1X PBS as scanning medium, as described previously [3]. The x-ray tube voltage was 55 kV and the current was 145 μA, with a 0.5 mm aluminium filter. The exposure time was 1180 ms.

### Histology

After obtaining μCT images, both grade I and grade IV samples were cut in two. Half the samples were processed by decalcification for histological observations and the other half were used for un-decalcified resin embedding for histomorphometric studies. For decalcified tissue processing, samples were demineralised (pH 7.4) in 10 % ethylene diamine tetraacetic acid (EDTA) for six weeks at 4 °C. They were then dehydrated through ascending alcohol concentrations and embedded in paraffin wax. Sections, 5 μm thick, were cut with a microtome and placed on 3-aminopropyltriethoxy-silane coated glass slides. Each specimen was stained with hematoxylin and eosin (H&E) to visualize tissue microstructure. For resin embedding, samples were fixed overnight in 2 % PFA and 2 % glutaraldehyde buffer at pH 7.4 with 0.1 M sodium cacodylate. The tissue specimens were dehydrated in ascending concentrations of ethanol (from 70 % to 100 %) and embedded in poly-methyl methacrylate (PMMA). For von Kossa stain, 30 μm sections of resin embedded samples were cut using an automated sledge microtome (Reichert-Jung, Polycut S) and collected onto gelatine coated microscope slides, which were covered with a plastic film and dried overnight at 60 °C. The plastic film was dissolved in xylene and the samples rehydrated and stained using von Kossa staining procedures as described previously [3].

### Back-scattered scanning electron microscopy analysis and focus ion beam prepared TEM specimen preparation

Ten resin embedded specimens (five grade I samples and five grade IV OA samples) were polished using 1 μm and 0.3 μm Alpha Micropolish Alumina II (Buehler) on a soft-cloth rotating wheel. A stereomicroscope (Leica M125) was used to identify and label the boundary between cartilage and bone regions, after which the polished surfaces were coated with gold-palladium and examined using FEI/Philips XL30 Field-Emission Environmental Scanning Electron Microscope, operating at 15 kV for back-scattered SEM (BSEM) observation. After obtaining BSEM images, a Dual Beam FEI Quanta 200 3D FIB system was used to prepare a TEM cross-sectional specimen; this system allows the accurate positioning of the subchondral bone plate region and subchondral trabecular bone region through its in situ “lift-out” technology [14]. The FIB was operated at low beam currents of 30 pA to 5 nA and an acceleration voltage of 30 kV. A piece of the specimen, containing the region of interest, was lifted out of the specimen block and positioned on a specially prepared half-grid with grid bars extending into the center of the copper FIB grid after FIB milling; after this the piece was thinned to approximately 100 nm by beam currents before further TEM evaluation.

### TEM imaging, electron diffraction, and elemental analysis

The FIB TEM ultrathin sections were observed by TEM (JEM-1400, JEOL, Japan) at an acceleration voltage of 100 kV. TEM images were photographed at high and low magnifications to fully capture the nanostructure features of the tissue. The diffraction patterns of the samples were recorded digitally using a selected-area aperture allowing observation of a circular area of 100 nm diameter. In situ Energy dispersive X-ray (EDS) analysis was also performed using 80 mm^2^ X-max Silicon Drift Detector (Oxford Instruments, UK). The Calcium-to-phosphate (Ca/P) ratios were calculated as the ratio between the atomic percentages of the two elements. Ca/P ratios were reported as averages ± standard deviation.

### Mineral extraction for HR-TEM and FTIR

The mineral extraction protocol was based on the previously published method by Mahamid et al. which reported that the protocol had little influence on alteration of the mineral phase [14]. In brief, freshly dissected OA knee bones were dissected into the grade I and grade IV regions as described above, after which the dissected bone was processed separately by freezing with liquid nitrogen and pulverised with a bead-beater machine. The bone powders were washed thoroughly with acetone to remove fatty tissue components and centrifuged at 10000 g for 2 min, after which the supernatants were removed. A 6 % sodium hypochlorite solution was added over 5 min at room temperature while the suspension was being stirred. The slurry was then centrifuged at 10000 g for 2 min to collect the pellet which was washed three times with Milli-Q water saturated with calcium and phosphate and then twice with 100 % ethanol. The pellet was resuspended in ethanol and sonicated with high intensity pulses for 1 min. A drop of the ethanol suspension was deposited on a carbon coated copper TEM grid and allowed to evaporate. A JEOL JEM-2100 TEM operating at 200 kV was used to capture images of the crystalline structure of the mineral particles. The dried mineral powders were also characterized with a Nicolet iS50 FTIR-ATR spectroscope (Thermo Scientific) with 32 scans at 4 cm^−1^ resolution in the absorbance mode. The spectra were then normalized to the intensity of the phosphate *ν*_1_, *ν*_3_ peak at 1012.59 cm^−1^. The splitting factor (SF) of the phosphate *ν*_4_ antisymmetric bending frequency at 550 – 605 cm^−1^ was calculated as the sum of the heights of the 558.65 and 600.03 cm^−1^ phosphate peaks, divided by the height of the trough between them [15, 16]. All heights were measured above a baseline drawn from approximately 440 to 700 cm^−1^. Calculated SF values were compared between the grade I and the grade IV bone mineral extracted from the same specimen. Five sets were measured from each sample.

### Nanoindentation analysis

Nanoindentation, as a measure of the nano-scale elastic and plastic response of bone, was used to evaluate the elastic modulus and hardness of bone [17, 18]. In this study, load-controlled nanoindentation measurements were performed using a TI 950 TriboIndenter (Hysitron, USA). A diamond Berkovich pyramidal indenter was used for all measurements under a trapezoidal loading function. The instrument was calibrated prior to testing using a standard fused quartz sample and standard aluminum sample. The constant loading time was 5 s and reached a maximum load (*P*_max_) of 2000 mN, which was followed by a dwell time of 2 s with the same load; the unloading phase was performed at the same rate as the loading phase. All measurements were performed on the same two microstructures of subchondral trabecular bone: lamellae and osteons. A total of 48 indentations were made in each structure at a minimum spacing of 5 μm between each indent in one specimen, both elastic modulus and hardness (*H*) of bone tissues were calculated from the unloading segment of the load–displacement curve according to the Oliver and Pharr method [19]. Elastic modulus is related to the stiffness of the bone, with a higher modulus being indicative of a stiffer material. Taking account of the elastic deformation that occurred in both sample and indenter tip, reduced modulus (*E*_*r*_) is represented as the elastic modulus of bone resin block by the following equation: (Eq. 1)

1 $$ \frac{1}{E_r}=\frac{\left(1\hbox{-} {\nu}^2\right)}{E}+\frac{\left(1\hbox{-} {\nu}_i^2\right)}{E_i} $$

where *ν* was Poisson’s ratio for the indented specimen, *ν*_*i*_ and *E*_*i*_ refered to the Poisson’s ratio and elastic modulus of the indenter material (*ν*_*i*_ = 0.07, *E*_*i*_ = 1440 GPa), respectively [20].

The *H* accounts for bone resistance to plastic deformation and has the normal definition: (Eq. 2)

2 $$ H=\frac{{\mathrm{P}}_{\max }}{A} $$

where *P*_*max*_ is the maximum indentation load and *A* is the projected contact area at that load [21]. Three sets of the grade I and the grade IV bone resin blocks were measured. Mean values for the *E*_*r*_ and *H* were calculated for each specimen.

### Statistics

The data were analyzed with IBM SPSS statistics software, version 22 (SPSS Inc., Chicago, IL, USA). One-way analysis of variance (ANOVA) followed by Student–Newman–Keuls-*q* (SNK-*q*) tests were performed for multiple comparisons, and paired *t*-test was using for comparisons of Ca/P ratio and the SF value of mineral crystals from grade I and grade IV trabecular bone. For all comparisons, the significance level was set at *α* = 0.05.

## Results

### Morphology and mineralization of the OA subchondral bone graded according to disease severity

**Fig. 1:**
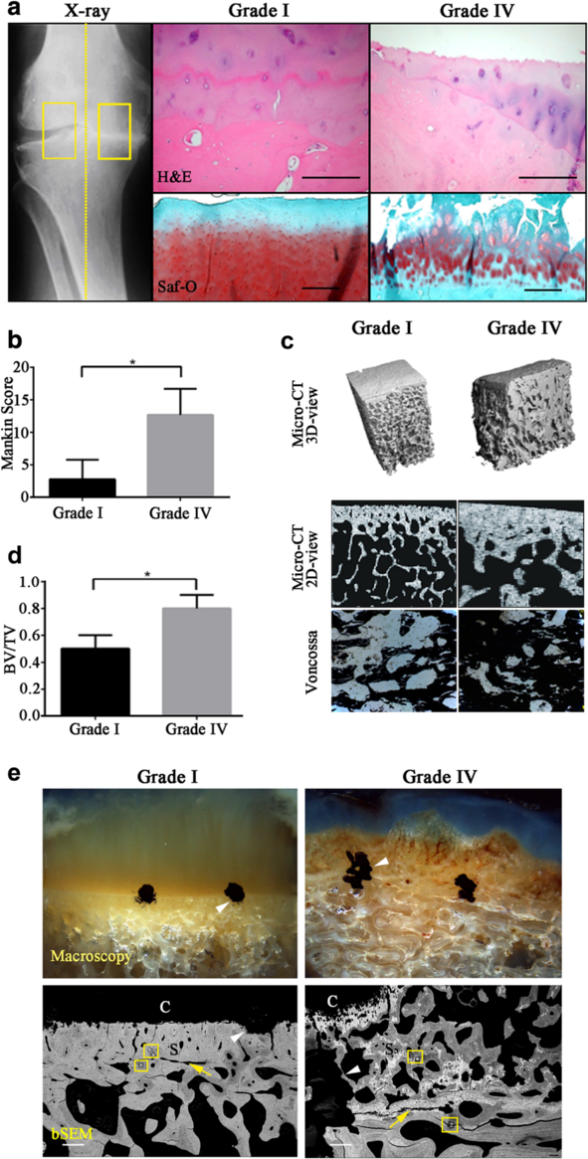
Representative X-ray, macroscopic histology, μCT and backscatter SEM images of OA samples. (**a**) X-ray showing joint space narrowing and the non-sclerotic and sclerotic region of OA subchondral bone; H&E and Safranin-O staining of OA samples graded according to the disease severity. (**b**) Mankin scoring was performed to assess the disease severity of grade 1 and grade IV samples. *N* = 10 separate samples. * P represents that the difference was statistically significant (*P* <0.05). (**c**) μCT images of OA samples graded according to disease severity, 3D and 2D view of grade I OA bone compared to grade IV OA bone. Von Kossa staining of grade I OA subchondral bone and grade IV OA bone. Scale bar = 100 μm. (**d**) Quantitative μCT results show grade IV subchondral bone plate has a higher bone volume fraction compared to grade I specimens. *N* = 5 separate samples. * P represents that the difference was statistically significant (*P* <0.05). (**e**) Resin embedded grade I OA samples showing a distinct boundary between articular cartilage and subchondral bone compared to grade IV OA samples and backscatter SEM images of grade I OA bone vs. grade IV OA subchondral bone. Black spots (white arrow heads) indicate the position of the dividing line between cartilage and bone. The border between subchondral bone plate and trabecular bone was distinguished by the bone plate arrangement (yellow arrows). TEM slices were “lifted out” of the representative positions in bone sample (yellow rectangles). Scale bar = 100 μm. C: articular cartilage; S: subchondral bone plate; T: trabecular bone

X-ray images of grade IV OA samples showed joint space narrowing (indicating a loss of articular cartilage), marginal osteophyte formation and subchondral bony sclerosis, which indicated an abnormal bone mineral density and disordered joint structure (Fig. 1a). H&E staining was performed to confirm the site specific changes in the samples (Fig. 1a). All grade I samples showed articular cartilage with a normal appearance of the underlying subchondral bone with a clearly defined tidemark. By contrast, grade IV OA specimens showed evidence of cartilage loss with a small region at the edge of the slide where there was some preservation of the deep and middle zone cartilage layers. Increased cartilage damage was also confirmed in grade IV samples by Safranin-O staining and increased Mankin scoring (Fig. 1a and b). Subchondral bone changes were detected in all OA specimens. Two-dimensional and three dimensional μCT scans revealed that grade IV OA subchondral bone was denser and thicker, without a clear border between bone plate and the trabecular bone (Fig. 1c) compared to grade I OA samples. Quantitative μCT data revealed that grade IV OA specimens had increased bone volume fraction compared to the control grade 1 group (*P* = 0.049) (Fig. 1c and d). Von Kossa staining showed an abnormally intense degree of staining in the grade IV region (Fig. 1c) indicating an excessive mineral deposition.

Viewed under low magnification, the resin embedded samples revealed distinct differences between grade I and IV OA. The well-defined tidemark between calcified and uncalcified tissue seen in grade I samples (Fig. 1e) was absent in the grade IV samples and instead replaced with a disordered morphology (Fig. 1e). BSEM data suggests a thickening of the grade IV subchondral bone plate and heterogeneous distribution of more highly mineralized tissue, seen as a bright phase (Fig. 1e). By contrast, the grade I bone showed homogeneous mineralization of both the subchondral bone plate and trabecular region (Fig. 1e). Collectively, these results suggest a site-specific changes in the severely affected subchondral bone of OA patients.

### Nano-structural properties of OA subchondral bone plate and subchondral trabecular bone

**Fig. 2:**
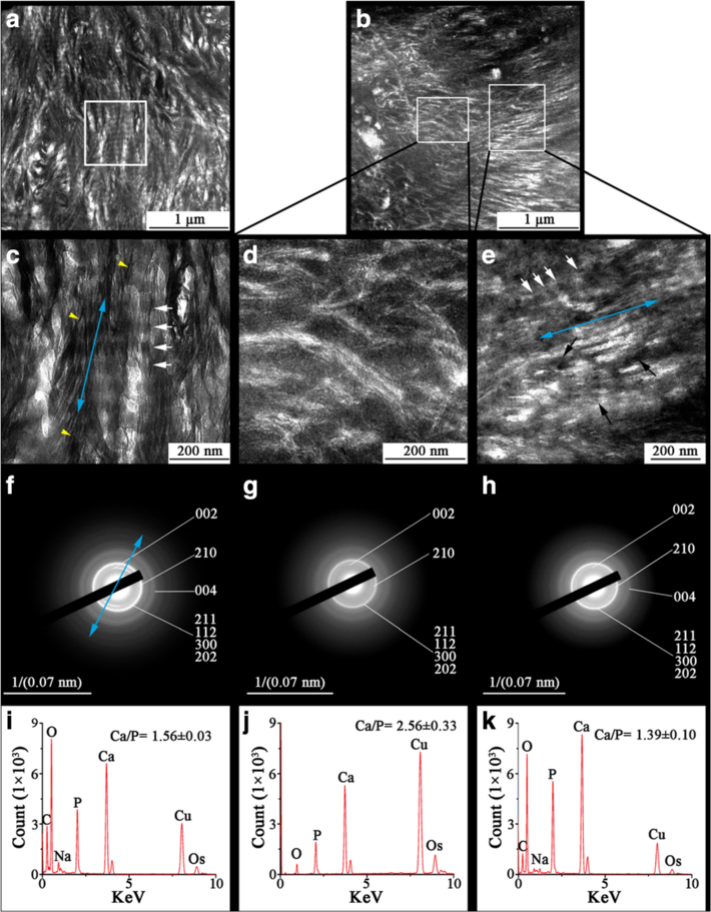
TEM and SEAD imaging of thin unstained sections from the grade I OA and the grade IV OA subchondral bone plate in correlation to EDS analysis. (**a**) Characteristic fibril banding patterns were seen in the grade I OA subchondral bone plate. (**b**) The grade IV bone showed an electron dense region lacking a hierarchal structure (left rectangle) combined with a cross-banding pattern in the remaining regions (right rectangle). (**c**) High magnification of the selected area in image "a" shows faint bands (white arrows) which were perpendicular to the long axis of fibril (blue arrow). Clusters of linear features with a distinct profile (yellow arrow head) could be seen. (**d**) High magnification “non-structured” region. (**e**) High magnification of the fibril-banding pattern region in the grade IV bone showed fibrils with amorphous darker bands (white arrows) and the intensified electron dense spread to whole fibril along with its long axis (blue arrow) which exhibited non-hierarchical structure (black arrows). (**f**) SEAD pattern of the grade I OA bone, blue arrow indicates the *c*-axis orientation of carbonated HA within the tissue. (**g**) SEAD pattern shows weakened diffraction of high density region in the grade IV OA bone. (**h**) SEAD pattern shows weakened diffraction of cross-banding patterned region in the grade IV OA bone. (**i**) EDS spectra of the grade I OA bone. (**j**) EDS spectra of the high density region in the grade IV OA bone. (**k**) EDS spectra for the cross-banding patterned region in the grade IV OA bone. The images are representative of 5 different patient samples graded according to the disease severity

**Fig. 3:**
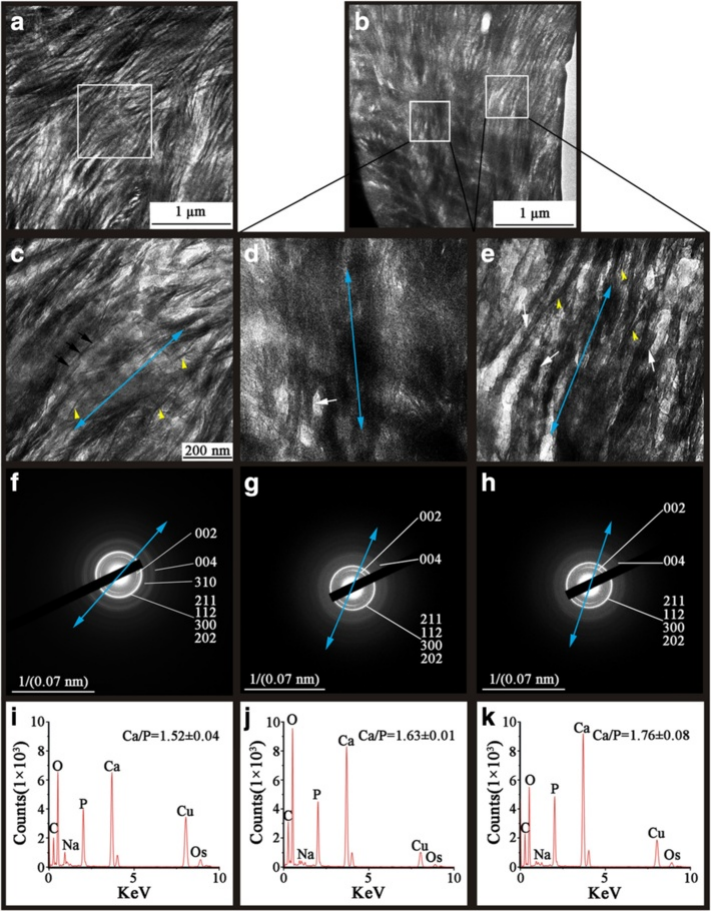
TEM and SEAD images of thin unstained sections from the grade I OA and the grade IV OA trabecular bone correlated with the corresponding EDS analysis. (**a**) Characteristic fibril banding patterns are seen in grade I OA trabecular bone. (**b**) The grade IV bone shows a region of high electron density lacking a hierarchal structure (left rectangle), combined with cross-banding patterns in the remaining regions (right rectangle). (**c**) High magnification of selected area in image "a" show faint bands (black arrows) which are perpendicular to the long axis of fibril (blue arrow). Clusters of linear features with a distinct profile can be seen (yellow arrow heads). (**d**) High magnification of a region with indistinct structure showed a possible long axis of fibrils (blue arrow) and completely mineralized fibrils (white arrows) next to the electron dense region. (**e**) High magnification of the cross-banding pattern region in the grade IV bone shows fibrils with wide darker bands (black arrows) and with increasing electron density spread to whole fibrils along the long axis (blue arrow) which exhibited non-hierarchical structures (white arrows); discrete dark features were identified with amorphous profile (yellow arrowheads). (**f**) SEAD pattern of the grade I OA bone, blue arrow indicates the preferential *c*-axis orientation of carbonated HA within the tissue. (**g**) SEAD pattern of high dense region in the grade IV OA bone, blue arrow indicated the predominant *c*-axis orientation of carbonated HA within the tissue. (**h**) SEAD pattern of cross-banding pattern region in the grade IV OA bone, blue arrow indicates the predominant *c*-axis orientation of carbonated HA within the tissue. (**i**) EDS spectra of the grade I OA bone. **(j)** EDS spectra of the high density region in the grade IV OA bone. (**k**) EDS spectra of the banding pattern region in the grade IV OA bone. The images are representative of 5 different patient samples graded according to the disease severity

The TEM images of the grade I subchondral bone plate (Fig. 2a) and subchondral bone trabecular bone (Fig. 3a) displayed a periodic fibril banding-like nanostructure typically observed in the normal bone. High magnification TEM images further showed ubiquitously elongated dark features running perpendicular to the periodic bands and, therefore, parallel to long axis of the fibril in grade I subchodral bone (Figs. 2c and 3c). However, the grade IV subchondral bone exhibited an altered architecture with uneven fibril alignments (Figs. 2b and 3b). In some regions of grade IV subchondral bone (Figs. 2e and 3e), the discrete dark features were not seen. These features coalesced to form intra-fibrillar mineral strands which led to an amorphous border between white band and dark band. Some mineralized fibrils lacked a banding pattern altogether. In contrast, some areas located in the same samples were replaced by a random, undulated arrangement (Figs. 2d and 3d) and the dense electron distribution suggesting bulk mineral aggregation.

A comparison of the diffraction patterns of grade I and grade IV subchondral bone plate with the corresponding SAED images captured from the specimens revealed that the *c* axis of the crystal lattice (defined as connection of the midpoints of 002 arc reflection) was parallel to the long axis of the fibrils (Fig. 2f). However, the orientation of the carbonated HA was absent in the grade IV subchondral bone plate (Fig. 2g and h), showing weakening diffraction pattern of the mineral crystal in the severely affected region of the grade IV subchondral bone plate, indicative of mismatched structure between fibrils and mineral crystals.

When comparisons were made between the diffraction patterns of subchondral trabecular bone, the grade I trabecular bone exhibited some pronounced concentric ring patterns that were indexed to the (002), (211), (112), (300), (202), (310) and (004) planes (Fig. 3f), suggestive of well-organized crystal distribution in this region. By contrast, in grade IV trabecular bone, the absence of (310) plane in grade IV trabecular bone was an indication of altered mineral crystal orientation in the *α* axis in this area (Fig. 3g and h). Despite the fibrils displaying a somewhat “cloudy” profile in the severely affected region of grade IV trabecular bone, their long axes could still be inferred from the general arrangement of fibrils. Unlike the axial consistency of fibrils and mineral crystals in grade I trabecular bone, the crystals in grade IV trabecular bone had a staggered direction relative to the orientation of fibrils (Fig. 3g), indicating a disordered nanostructure of the bone.

Stoichiometric analyses were further conducted by *in situ* EDS which showed heterogeneous distribution of the average Ca/P ratio in OA bone sample (Additional file 1: Tables S1 and S2). Compared to the grade I bone, the severely affected OA region of grade IV bone had a higher Ca/P ratio (Figs. 2j and 3j) (*P* < 0.05). The ratio of the less affected OA regions in the subchondral bone plate had a lower value than that of the grade I sample (Fig. 2k) (*P* < 0.05), whereas the less affected OA region in trabecular bone had a higher Ca/P ratio than that of both the grade I and severely affected OA regions (*P* < 0.05) (Fig. 3k).

### Mineralization properties OA subchondral bone

**Fig. 4:**
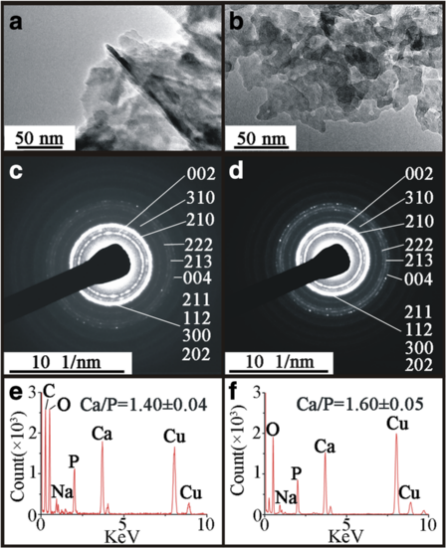
HR-TEM and SAED correlated with EDS spectrum images of freshly extracted minerals from grade I (**a, c, e**) and grade IV OA sourced trabecular bone (**b, d, f**). (**a, b**) Nano-particles of minerals extracted from (**a**) grade I and (**b**) grade IV OA trabeculae. (**c, d**) SAED pattern of mineral particles from (**d**) grade IV OA trabeculae exhibited higher intensity diffraction rings than the (**c**) grade I OA trabeculae. (**e, f**) EDS spectra for the nano-mineral particles from the (**e**) grade I and the (**f**) grade IV OA trabeculae. The images are representative of 5 different patient samples graded according to the disease severity

Although nano-minerals extracted from grade I and grade IV OA trabeculea were found to share quite similar morphology (Fig. 4a and b), subtle distinctions between them were identified by SAED, EDS and FTIR.

The SAED patterns produced by the grade IV OA trabecular minerals had sharper diffraction rings, especially the planes of (222), (213) and (004) (Fig. 4d) compared with the grade I OA trabeculea, bone in which the (222), (213) and (004) planes appeared as diffuse rings (Fig. 4c) indicating greater mineral crystallinity in grade IV OA trabeculae.

The EDS elemental analyses of mineral crystals from grade IV bone revealed a higher average Ca/P ratio (*P* < 0.05) (Fig. 4f) compared with grade I OA trabeculae (Fig. 4e) (Additional file 1: Table S3).

**Fig. 5:**
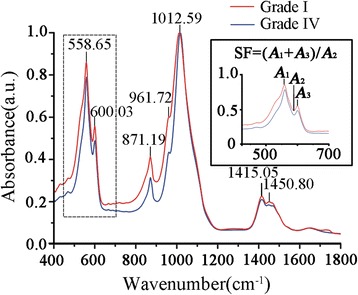
FTIR-ATR spectra of freshly extracted mineral particles from the grade I and the grade IV OA trabecular bone. The splitting factor (SF) was calculated by the formula inset. The images are representative of 5 different patient samples graded according to the disease severity

FTIR spectra of minerals extracted from the grade I and grade IV OA trabeculae were characteristic of carbonated HA (Fig. 5), as shown in other studies [14, 22]. The band at 1012.59 cm^−1^ corresponded to symmetric phosphate ν_1_, ν_3_ absorbance. Compared to the grade IV OA trabecular mineral, the grade I OA trabucular minerals presented a border band at this peak, as well as higher pronounced peaks at 872, 961.72, 1415.05 and 1450.80 cm^−1^ which corresponded with carbonate absorption. The minerals from the grade IV OA trabeculae consistently produced higher SF values (3.72 ± 0.08) compared to the grade I OA bone (3.30 ± 0.15) (*P* < 0.05) (Fig. 5, inset) (Additional file 1: Table S4). The FTIR spectra was indicative of increased crystallinity of mineral particles taking place within the grade IV OA trabeculae, lower crystallinity of grade I OA trabeculae may explained by the increased carbonate absorption. Altered mineral crystallinity may contribute to the altered mechanical properties in OA bone.

### Mechanical properties of OA subchondral bone plate

**Fig. 6:**
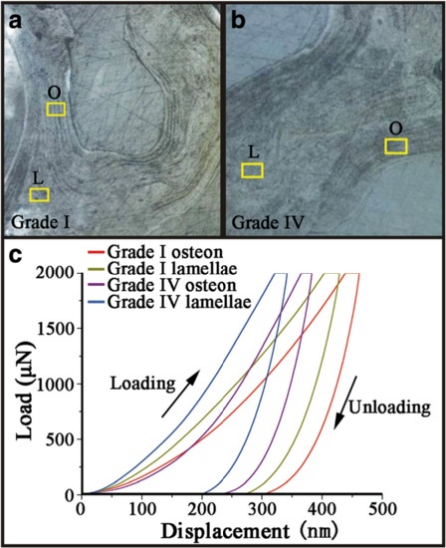
**(a** and **b**) show the microstructures in polished bone samples. O: osteon; L: lamellae. (**c**) shows the load–displacement curve of bone samples. The images are representative of 10 different patient samples graded according to the disease severity

The microstructures of the polished surface of the trabeculae included lamellae and osteons, distinguished by the presence of a vessel channel at the center of the osteon (Fig. 6a and b). It was found that lamellae in both grade I and grade IV trabeculae exhibited a shorter displacement than osteon under a constant force and loading rate (Fig. 6c). All the nano-indentation curves were relatively smooth without discontinuities. The intrinsic bone tissue mechanical properties (summarized in Table 1) showed increased *E*_r_ and *H* values (19 % and 20 %) in the grade IV OA trabecular osteon compared to the grade I OA osteon (*P* < 0.05). Furthermore, it also showed similar increases in *E*_r_ and *H* values of 25 % and 17 % in the grade IV OA trabecular lamellae (*P* < 0.05).

**Table 1:** Average elastic module and hardness values of resin, the grade I and the grade IV trabecular bone. Values (mean ± SD) with different superscript letters (a vs b vs c) and different superscript symbols (* vs △ vs □) in the same row were significant difference (one-way ANOVA analysis and SNK-*q* test, *P* < 0.05). E: Elastic modulus; H: Hardness

	Resin	Osteon		Lamellae	
		Grade I	Grade IV	Grade I	Grade IV
E (GPa)	3.35 ± 0.23^a,^*	13.46 ± 2.41^b^	16.00 ± 2.60^c^	13.90 ± 2.75^△^	17.33 ± 3.13^□^
H (GPa)	0.16 ± 0.02^a,^*	0.46 ± 0.12^b^	0.55 ± 0.14^c^	0.53 ± 0.14^△^	0.62 ± 0.10^□^

## Discussion

We hypothesized that OA bone changes were related to changes to the physicochemical properties of bone materials and not simply changes to overall bone mass. To test this hypothesis we analysed subchondral bone from the superior and inferior sectors of tibial sections from OA patients. The results from this study demonstrated a relationship between the pathological changes in OA bone and changes to mineral phase of the bone at the nano-structural level.

It is widely accepted that bone stiffness and ductility are strongly influenced by the collagen fibers and the physiochemical property of carbonated HA, respectively [23, 24]. In this study, we observed that the fibrilar skeleton lost its well-organized appearance in the severely affected subchondral bone plate and sclerotic trabeculae (Figs. 2d and 3d) in grade IV bone (Figs. 2e and 3e). Moreover, grade IV bone adjacent to the severely affected lesion also displayed non-hierarchical intra-fibrillar mineralization; however, the Ca/P ratios showed some kind of crosscurrent distribution in subchondral bone plate and trabeculae. Increasing electron density in grade IV bone was suggestive of mineral aggregation which could result in the fibrils having less ductility and being subjected to greater compressive stress [25]. The subchondral bone plate lies immediately beneath the calcified zone of the articular cartilage. Due to its anatomical position, the subchondral bone plate carries most of the load passing through the joint. It is therefore under constant stress and consequently has a high rate of metabolism [26], which could alter the distribution of calcium ion and the form of bony salts. Consistent with our observations, Buchwald et al. [27] observed the ratio of carbonate apatite to hydroxyapatite is higher in the subchondral bone plate from OA patients, which indicated deficient mineralization and had an impact on mineral crystal growth [28]. In another study, it has been reported that bone from the iliac crest have higher mineral contents by density fractionation of cortical bone and back scattered electron microscopy. These data indicate that site and bone type may be important factors governing the changes caused by OA. The high frequency of bone turnover in OA subchondral bone plate leads to an unstable environment for lesion recovery and normal mineralization, and failure to form crystalline and correctly oriented mineral crystals. Deterioration of subchondral bone plate structure could expose subjacent trabeculae to abnormal mechanical stresses and thus caused pathological and adaptive changes in trabeculae [29]. Changes in collagen could also affect the mineralization process [30]. The arrangement of fibrils could alter the way that collagen molecules interact with each other and with surrounding macromolecules and would, therefore, ultimately affect the morphology and arrangement of minerals formed in the collagen matrix [30]. The diffused 002 planes in the grade IV subchondral bone plate suggest changes to the orientation and phase of mineral crystals (Fig. 2g and h). In the severely affected lesion of grade IV bone (Fig. 3h), non-parallel arrangement of fibrils and mineral crystals could cause an abnormal load transmission and makes it hard for fibers to dissipate the deformation energy and thus promote the micro-damage. Nano-sized mineral crystals showed a highly ductile behavior, but at the macro-scale were increasingly brittle [31]. We made the observation by TEM that fibrils in sclerotic subchondral bone plates and trabeculae undergo changes to mineralization and rearrangements, something which highlights the complex pathological mechanism of OA disease. The amorphous profile of mineral crystals in sclerotic trabeculae is indicative of a coalescence of mineral particles that is reported to lead increased bone brittleness.

Disordered arrangement of organic and inorganic composition had a negative effect on load transmission [32]. Distinctly heterogeneous distribution of Ca/P ratios in subchondral bone plate is adverse to form a proper mineral phase that could absorb load stresses, conversely, more abnormal stresses were transmit to subjacent trabeculae. Bone strength and stiffness increased with increasing mineral crystallinity [31]. As a compensatory reaction, increased mineral crystallinity in trabeculae could be a possible explanation to the increment of localized stress absorption. Varying Ca/P ratio affected mineral crystals in the physical, mineralogical and mechanical characteristics [28, 33]. Compared with the grade I tabeculae, Ca/P ratio in grade IV trabeculae showed an uneven distribution (Fig. 3). The ratio could be affected by organic phosphate in tissue. To eliminate this possibility, we extracted the mineral ingredients from the fresh trabeculea. The plate-like structure in OA trabeculae has been reported [8, 34], which reflected high mechanical stress and was associated with similar morphology of mineral crystals as shown in this study (Fig. 4b) [35]. The crystals from the grade IV OA trabeculae produced sharper diffraction rings by electron diffraction plus Ca/P ratio values approaching the theoretical value of HA, suggesting a possibly higher crystallinity. This notion was supported by the changes in the infrared splitting factor, the value of which showed a numerical increase in the mineral crystals from sclerotic trabeculae. Increased mineral crystallinity and non-hierarchical intra-fibrillar mineralization in subchondral sclerosis would further enhance the localized stiffness of bone material and lead to a corresponding absorption of local stresses, the non-affected region near the lesion could suffer from the atrophy of disuse and thus display localized stress shielding, evidenced by lower Ca/P ratio in grade I bone [36]. This opens up to the possibility of a “mineralization adaptation zone” between the lesion area and non-affected area, which would assist the localized load transmission and lead to increased subchondral sclerosis [37]. Excessive intra-fibrillar mineralization not only increases the intra-fibrillar density of mineral content but also contains minerals with higher crystallinity. However, at some point high carbonated HA crystallinity is associated with bone brittleness, which implies that the more crystalline the bone the more liable it is to form critical sized cracks, since it is less able to withstand deformation [38]. It is worth noting that technical limitations make it impossible to extract the mineral crystals from the subchondral bone plate.

Previous studies have revealed that structural changes to OA bone is the result of mechano-regulated bone adaptation [39, 40]. In the sclerotic trabeculae, pathological remodeling of the bone results in disordered fibrils and mineral crystal arrangements. In the present study, the grade IV OA trabeculae obtained a higher *E*_r_ and *H* values as OA progresses, whereas disordered structure and high crystalline mineral content made grade IV OA bone less tolerant to micro-cracks of the order of several hundred micro-meters, a size which may be essential for normal bone remodeling. During the active OA stages, the mineral deposition is attenuated in the lesion region by high bone turnover, resulting in hypomineralization of the bone [41]. This is associated with less stiffness and causes the bone structure to collapse more readily under load. Micro-cracks were generated and healed to form a thicker and denser subchondral bone structure for mechanical adaptation. However, the healing progresses were depressed due to low bone turnover at the late OA stage and thus produced more micro-cracks in the sclerotic lesion [41, 42].

Altered anisotropic mechanical properties were found between the grade I and grade IV OA regions, which may increase the bone brittleness, thus leading to macroscopic failure of the tissue and the risk of catastrophic bone fractures. When the mechano-regulatory pathway of bone is activated [39], the continued deposition of minerals may lead to a localized hyper-mineralized phase of the subchondral bone during the OA stationary stage, and low bone turnover in the lesion region results in an abnormal aggregation of mineral crystals in the sclerotic region. This creates a stable micro-environment for mineral crystallization and an increased *E*_*r*_ value, which, is in turn, compels the bony stiffness to deal with more force. Paradoxically, the ductility of bone is suppressed by non-hierarchical intra-fibrillar mineralization and high crystalline mineral crystal, showing a higher *H* value, which is indicative of high bone brittleness in OA. Both increments of the *E*_r_ and *H* values in grade IV trabeculae indicated that both osteons and lamellae were subject to significant changes in mechanical properties during OA disease progression. A higher modulus increases resistance to elastic deformation and an increased hardness accounted for the stiff but brittle properties of bone. This supports the notion that sclerotic trabecular bone had a denser structure and stiffer property than the grade I OA trabecular bone that had suffered osteoporosis [43]. Furthermore, increased crystallinity of the mineral phase increases the chemical stability of the crystals [44] and leads to reduced rates of bone turnover in sclerosis and, therefore, results in stiffer bone material property than the grade I trabeculae. Using micro-indentation testing and electron probe microanalysis of the hip, Coats et al. has shown a reduced hardness and elastic modulus in OA bone when compared to osteoporotic bone [45]. However, in our study we found an increase in the hardness and elastic modulus compared to mild OA bone. These differences could be attributed to the location of the sample site differences between hip and knee. In another study, Li et al., showed an altered mechanical and material properties of the subchondral bone plate from the femoral head of patients with either OA or osteoporosis [46, 47]. Our data add a novel perspective to the general understanding of the bone stiffening mechanism in subchondral sclerosis. It is well-documented that sclerotic bone has less mineralization in the lesion region [6] but absorbs the most stress of the bone [43], and in this study we observed that hypomineralized subchondral sclerosis displayed a disordered mineralization distribution and that hypermineralized parts in trabeculae could assist with localized stress absorption. Increased intra-fibrillar mineral density also results in the fibrils having less ductility and being subjected to greater compressive stress. Furthermore, increased crystallinity of the mineral phase renders higher stiffness to bone, and increased chemical stability of the crystals leads to reduced rates of bone turnover in sclerosis [44].

## Conclusion

Here, we have presented evidence of nano-structural differences between the OA grade I and the grade IV subchondral bone, which provides new insights into the basis of bone fragility, a characteristic feature of OA. Excessive intra-fibrillar mineralization could account for lower ductility of the collagen network. Moreover, the presence of the highly crystallized calcium-phosphate phases in grade IV bone may account for the sclerotic characteristics of the bone in these regions, which results in an altered response to load transmission and thus leads to cartilage degeneration.

## Additional file

Additional file 1: Table S1. EDS data of subchondral bone plate. Values (mean ± SD) with different superscript letters (a vs b vs c) were significant difference (one-way ANOVA analysis and SNK-*q* test, *P* < 0.05). **Table S2.** EDS data of trabecular bone. Values (mean ± SD) with different superscript letters (a vs b vs c) were significant difference (one-way ANOVA analysis and SNK-*q* test, *P* < 0.05). **Table S3.** The EDS data of mineral crystals are shown as follow. Values (mean ± SD). Significance = *P* < 0.05. **Table S4.** The splitting factor data of mineral crystals are shown as follow. Values (mean ± SD). Significance = *P* < 0.05. (DOC 104 kb)

## Abbreviations

BMD: Bone mineral density
BSEM: Back-scattered SEM
Ca/P: Calcium (Ca) to phosphorous (P) ratios
COL I: Type I collagen
EDS: Energy dispersive X-ray
EDTA: Ethylene diamine tetraacetic acid
FTIR: Fourier transform infrared spectroscopy
HA: Hydroxyapatite
MRI: Magnetic resonance imaging
OA: Osteoarthritis
PFA: Paraformaldehyde
PMMA: Poly-methyl methacrylate
TEM: Transmission electron microscopy
μCT: Micro-computer tomography
