# Supplementary material for: Characterization of nano-structural and nano-mechanical properties of osteoarthritic subchondral bone
Source: BMC Musculoskelet Disord. 2016 Aug 24;17(1):367. doi: 10.1186/s12891-016-1226-1 (PMC4997740; doi:10.1186/s12891-016-1226-1)
Supplement: Additional file 1: Table S1. — EDS data of subchondral bone plate. Values (mean ± SD) with different superscript letters (a vs b vs c) were significant difference (one-way ANOVA analysis and SNK-q test, P < 0.05). Table S2. EDS data of trabecular bone. Values (mean ± SD) with different superscript letters (a vs b vs c) were significant difference (one-way ANOVA analysis and SNK-q test, P < 0.05). Table S3. The EDS data of mineral crystals are shown as follow. Values (mean ± SD). Significance = P < 0.05. Table S4. The splitting factor data of mineral crystals are shown as follow. Values (mean ± SD). Significance = P < 0.05. (DOC 104 kb) [file 12891_2016_1226_MOESM1_ESM.doc]

**Supplementary Data:**

**Supplementary Data Table S1:** EDS data of subchondral bone plate

| Sample | n | Ca | P | Ca/P ratio | mean ± SD |
| --- | --- | --- | --- | --- | --- |
| Grade I | 1 | 10.73 | 6.86 | 1.56 | 1.56±0.03^a^ |
|  | 2 | 11.28 | 7.23 | 1.56 |  |
|  | 3 | 11.02 | 6.83 | 1.61 |  |
|  | 4 | 13.21 | 8.61 | 1.53 |  |
|  | 5 | 10.83 | 7.07 | 1.53 |  |
| OA Grade IV severely affected area | 1 | 60.78 | 27.79 | 2.19 | 2.56±0.33^b^ |
|  | 2 | 65.28 | 23.13 | 2.82 |  |
|  | 3 | 64.79 | 24.34 | 2.67 |  |
|  | 4 | 55.76 | 25.04 | 2.23 |  |
|  | 5 | 64.14 | 22.18 | 2.89 |  |
| OA Grade IV less affected area | 1 | 17.44 | 11.16 | 1.56 | 1.39±0.10^c^ |
|  | 2 | 17.39 | 13.37 | 1.30 |  |
|  | 3 | 14.54 | 10.84 | 1.34 |  |
|  | 4 | 16.54 | 11.64 | 1.42 |  |
|  | 5 | 16.43 | 12.28 | 1.34 |  |

Values (mean ± SD) with different superscript letters (a vs b vs c) were significant difference (one-way ANOVA analysis and SNK-*q* test, *P* < 0.05)

**Supplementary Data Table S2:** EDS data of trabecular bone

| Sample | n | Ca | P | Ca/P ratio | mean ± SD |
| --- | --- | --- | --- | --- | --- |
| Grade I | 1 | 13.18 | 8.7 | 1.51 | 1.52±0.04^a^ |
|  | 2 | 12.92 | 8.17 | 1.58 |  |
|  | 3 | 14.51 | 9.69 | 1.50 |  |
|  | 4 | 12.65 | 8.54 | 1.48 |  |
|  | 5 | 12.16 | 8.07 | 1.51 |  |
| OA Grade IV severely affected area | 1 | 12.03 | 7.42 | 1.62 | 1.63±0.01^b^ |
|  | 2 | 10.75 | 6.58 | 1.63 |  |
|  | 3 | 13.45 | 8.3 | 1.62 |  |
|  | 4 | 16.87 | 10.18 | 1.66 |  |
|  | 5 | 13.45 | 8.22 | 1.64 |  |
| OA Grade IV less affected area | 1 | 12.35 | 6.79 | 1.82 | 1.76±0.08^c^ |
|  | 2 | 13.46 | 8.19 | 1.64 |  |
|  | 3 | 15.35 | 8.3 | 1.85 |  |
|  | 4 | 17.58 | 10.18 | 1.73 |  |
|  | 5 | 17.13 | 9.71 | 1.76 |  |

Values (mean ± SD) with different superscript letters (a vs b vs c) were significant difference (one-way ANOVA analysis and SNK-*q* test, *P* < 0.05)

**Supplementary Data Table S3:** The EDS data of mineral crystals are shown as follow:

| Sample | n | Ca | P | Ca/P ratio | mean ± SD | Two independent sample t-test (*P* value) |
| --- | --- | --- | --- | --- | --- | --- |
| Grade I | 1 | 15.6 | 11.12 | 1.40 | 1.40 ± 0.04 | *P* =0.000062 |
|  | 2 | 8.06 | 6.07 | 1.33 |  |  |
|  | 3 | 15.17 | 10.82 | 1.40 |  |  |
|  | 4 | 12.79 | 8.98 | 1.42 |  |  |
|  | 5 | 14.27 | 9.91 | 1.44 |  |  |
| Grade IV | 1 | 15.69 | 9.54 | 1.64 | 1.60 ±0.05 |  |
|  | 2 | 19.35 | 12.41 | 1.56 |  |  |
|  | 3 | 11.55 | 7.43 | 1.55 |  |  |
|  | 4 | 14.6 | 8.97 | 1.63 |  |  |
|  | 5 | 18.39 | 11.25 | 1.63 |  |  |

**Supplementary Data Table S4:** The splitting factor data of mineral crystals are shown as follow:

| Sample | n | Splitting factor | mean ± SD | Two independent sample t-test (*P* value) |
| --- | --- | --- | --- | --- |
| Grade I | 1 | 3.13 | 3.30± 0.15 | *P* =0.000554 |
|  | 2 | 3.35 |  |  |
|  | 3 | 3.42 |  |  |
|  | 4 | 3.44 |  |  |
|  | 5 | 3.15 |  |  |
| Grade IV | 1 | 3.64 | 3.72 ±0.08 |  |
|  | 2 | 3.81 |  |  |
|  | 3 | 3.72 |  |  |
|  | 4 | 3.63 |  |  |
|  | 5 | 3.79 |  |  |
